# Effect of infiltrating nasal packing with local anesthetics in postoperative pain and anxiety following sinonasal surgeries: a systemic review and meta-analysis^☆^

**DOI:** 10.1016/j.bjorl.2019.12.011

**Published:** 2020-02-12

**Authors:** Shao-Chen Tsai, Ming-Tang Lai, Yi-Lin Kao, Chia-Che Wu

**Affiliations:** a Taipei Medical University, Wang Fang Hospital, Department of Otorhinolaryngology, Taipei, Taiwan; b Taipei Medical University, School of Medicine, Department of Otorhinolaryngology, Taipei, Taiwan

**Keywords:** Sinus, Nose, Surgery, Pain, Packing, Seio, Nasal, Cirurgia, Dor, Tamponamento

## Abstract

**Introduction:**

Packing of the nasal cavity has traditionally been used for postoperative bleeding control and decreasing synechia formation in patients undergoing nasal surgeries. Although absorbable nasal packing has been gaining popularity in the recent years, nonabsorbable nasal packing is still often used in nasal surgeries in various parts of the world. It is known to be associated with pain and discomfort especially upon and during removal, and previous reviews have only evaluated the effects of local anesthetic infiltration of nasal packing in septal surgeries.

**Objective:**

To evaluate the effect of infiltrating nasal packing with local anesthetics in postoperative pain and anxiety following sinonasal surgeries

**Materials and methods:**

We searched the PubMed and Embase databases from their earliest record to April 27, 2019, randomized controlled trials and prospective controlled trials for review, and included only randomized controlled trials for data analysis. We included studies using topical anesthetics-infiltrated nasal packing following sinonasal surgeries and evaluated the effectiveness compared to placebo packing in pain reduction during postoperative follow up, as well as the effectiveness in anxiety reduction.

**Results:**

Among 15 studies included for review, 9 studies involving 765 participants contributed to the meta-analysis. In terms of pain reduction, our analysis showed significant standard mean differences regarding effectiveness at postoperative 1, 12, 24 h interval for all surgical groups combined, in the sinus surgery group, as well as during nasal packing removal. There was no consistent evidence to support the effectiveness in anxiety reduction.

**Conclusions:**

Our study supports anesthetics infiltration of nasal packing as an effective method in managing pain in patients with nasal packing after sinonasal surgeries. However, the level of evidence is low. More high-quality randomized controlled trials are needed to establish its effectiveness in reducing anxiety. We believe this review is of great clinical significance due to the vast patient population undergoing sinonasal surgeries. Postoperative local hemorrhage remains the greatest concern for ear nose and throat surgeons due to the rich vasculature of the nose and sinuses. Sinonasal packing provides structural support and serves as an important measure for hemostasis and synechia formation. Although absorbable packing has been gaining popularity in the recent years, nonabsorable packing materials are still used in many countries due to lower cost. Infiltration of nasal packing with local anesthetic provides a solution to the discomfort, nasal pressure and nasal pain experienced commonly by the patients as evidenced by our analysis.

## Resumo

**Introdução:**

O tamponamento da cavidade nasal tem sido usado tradicionalmente para controle do sangramento pós-operatório e diminuição da formação de sinéquia em pacientes submetidos a cirurgias nasais. Embora o tamponamento nasal absorvível tenha ganhado popularidade nos últimos anos, o tampão nasal não absorvível ainda é frequentemente usado em várias partes do mundo. Sabe-se que o tamponamento está associado a dor e desconforto, especialmente na sua remoção, e revisões anteriores avaliaram apenas os efeitos do tampão com anestésico local em cirurgias do septo nasal.

**Objetivo:**

Avaliar o efeito do tamponamento nasal infiltrado com anestésicos locais na dor e ansiedade pós-operatórias após cirurgias nasosinusais.

**Material e método:**

Para a revisão, pesquisamos nos bancos de dados PubMed e Embase desde o registro mais antigo até 27 de abril de 2019, incluímos ensaios clínicos controlados e randomizados, a ensaios clínicos prospectivos controlados e apenas ensaios clínicos controlados e randomizados para análise de dados. Incluímos estudos que usaram tamponamento nasal infiltrado com anestésicos tópicos após cirurgias nasosinusais e avaliamos a eficácia em comparação com o tamponamento com placebo na redução da dor durante o acompanhamento pós-operatório, bem como os efeitos na redução da ansiedade.

**Resultados:**

Entre os 15 estudos incluídos, 9, que envolveram 765 participantes, contribuíram para a metanálise. Em termos de redução da dor, nossa análise mostrou diferenças médias padrão significantes em relação à eficácia no pós-operatório nos intervalos de 1, 12, 24 horas para todos os grupos cirúrgicos combinados, no grupo da cirurgia sinusal e durante a remoção do tamponamento nasal. Não houve evidências consistentes para apoiar a eficácia na redução da ansiedade.

**Conclusões:**

Nosso estudo apoia o uso de tamponamentos nasais infiltrados com anestésicos locais como um método eficaz no tratamento da dor em pacientes após cirurgias nasosinusais. No entanto, o nível de evidência é baixo. São necessários mais ensaios clínicos randomizados de alta qualidade para estabelecer sua eficácia na redução da ansiedade. Acreditamos que esta revisão seja de grande significado clínico devido à vasta população submetida a cirurgias nasosinusais. A hemorragia local pós-operatória continua a ser a maior preocupação para os cirurgiões otorrinolaringológicos devido à rica vasculatura do nariz e seios nasais. O tamponamento nasosinusal fornece suporte estrutural e serve como uma medida importante para a hemostasia e formação de sinéquias. Embora o tamponamento absorvível tenha ganhado popularidade nos últimos anos, os materiais de tamponamento não absorvíveis ainda são usados em muitos países devido ao menor custo. A infiltração do tamponamento nasal com anestésicos locais fornece uma solução para desconforto, pressão e dor nasal comumente referida pelos pacientes, como evidenciado por nossa análise.

## Introduction

Packing of the nasal cavity has traditionally been used for postoperative bleeding control and decreasing synechia formation in patients undergoing nasal surgeries. Evidence provided by recent meta-analysis studies however, has not consistently demonstrated the benefits of nasal packing, either absorbable or non-absorbable, in various nasal surgeries.^1^, ^2^, ^3^ Nasal packing therefore, is not currently routinely recommended in all nasal surgeries, and is largely used at the discretion of individual surgeons.^4^

Despite the inconsistent evidence regarding its effectiveness, nasal packing is still often used in nasal surgeries in various parts of the world. Due to the significant nasal pain often associated with the use of traditional nasal packing, various pharmacological or physical agents, novel packing devices, or adjustments to application timing have been explored in the attempt to combat post-operative discomfort and aid recovery.^5^, ^6^ A review in 2016 investigated the role of perioperative local anesthetics for reducing pain following septal surgery, providing a low level of evidence that adding anesthetic agents to nasal packing decreased post-operative pain compared to nasal packing alone.^7^ However, only a limited number of studies were used for analysis, and no information was provided regarding sinus or other types of nasal surgeries. Therefore, we conducted a systemic review and meta-analysis on infiltrating nasal packing with topical anesthetics following sinonasal surgeries and aimed to evaluate its effectiveness on reducing postoperative pain and anxiety compared with nasal packing alone, as well as its effectiveness on pain and anxiety upon nasal packing removal.

## Methods

This study was reported in accordance with the PRISMA guidelines.

### Searching and selection strategy

This meta-analysis included randomized controlled trials and prospective controlled trials published in peer-reviewed journals in the English language. We focused on studies evaluating the effects of nasal packing infiltrated with topical anesthetic, whether in solution or ointment form, in patients receiving sinonasal surgeries. Studies involving direct injections of anesthetics into the nasal cavity were excluded. Studies were eligible if they compared the treatment effects of nasal packing infiltrated with topical anesthetic with placebo nasal packing and evaluated pain and/or anxiety level at various postoperative hours. Intraoperative local anesthetics and postoperative analgesic medications were allowed if they were arranged in the same condition for all groups.

We searched for relevant articles in the PubMed and Embase databases from their earliest record to April 27, 2019. Main search terms were “((((((nasal OR turbin* OR sinus* OR septum OR septal OR septoplasty OR FESS OR ESS OR surgery)))) AND ((analgesic* OR anesthe* OR local))) AND ((pack) OR packing)) AND (pain OR anxiety)”. (See Supplementary file for details). The Cochrane Library was scrutinized for relevant reviews for additional references.

### Quality assessment

Two reviewers (Shao Chen Tsai and Chia Che Wu) were responsible for assessing the quality included studies. We assessed the level of evidence using the GRADE system. The risk of bias was assessed by the Cochrane ‘Risk of bias’ tool in Review Manager Software 5.3, which consisted of seven items regarding random sequence generation, allocation concealment, blinding of participants and personnel, blinding of outcome assessment, incomplete outcome data, selective reporting, and other sources of bias. Each item was evaluated and classified as low, high, or unclear risk of bias for each included study. Discrepancies between reviewers at any stage were resolved through discussion and consensus.

### Outcome assessments

We investigated the treatment effects of the experimental interventions on pain at the 1st, 12th, and 24th hour after surgery, as well as the effects on anxiety level and pain reduction upon nasal packing removal. We also investigated pain at the 1st, 12th, and 24th hour after surgery specifically in studies involving sinus surgeries. We prioritized Visual Analogue Scale (VAS) as our outcome of choice for pain. Other continuous pain scales such as the Numeric Rating Scale (NRS) or Verbal analog scale were used if VAS was not documented. Anxiety was measured using the Hamilton Anxiety Scale or Ramsay Sedation Scale (RSS). Qualitative assessments of postoperative bleeding as well as reported adverse events were performed among the included studies.

### Data extraction

We extracted relevant data from each study with a standard data recording form. The means and Standard Deviations (SDs) of outcomes regarding pain level and anxiety at the above-mentioned follow-up interval were extracted. If included studies used various topical anesthetic agents in more than one experimental group or involved multiple surgical methods, we synthesized the combined means and SDs of outcomes (see supplementary files for the equations). If studies did not provide complete data for analysis, we contacted the corresponding authors via email to obtain the complete data.

### Meta-analysis

Our meta-analysis adheres to the comparison principle, “topical anesthetics-infiltrated nasal packing vs placebo nasal packing”. The Standard Mean Differences (SMDs) were obtained to assess the effect size. A random-effect model was used and a point estimate with a 95% Confidence Interval (95% CI) was presented. Heterogeneity across studies was tested using the I^2^ test. An I^2^ score of >50% indicated significant heterogeneity. The meta-analysis was performed using Review Manager Software 5.3.

## Results

**Figure 1:**
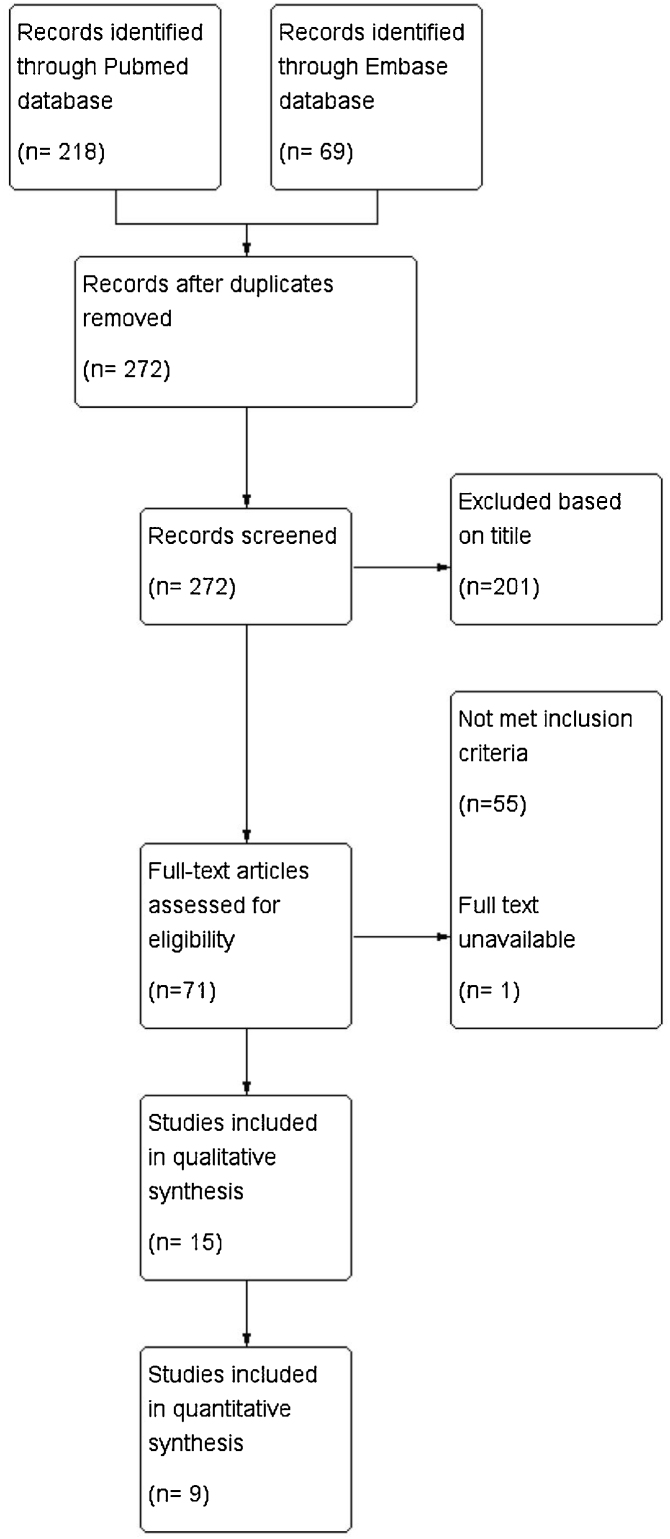
Flow of study diagram.

Our searches yielded 272 non-duplicated records. After exclusion based on the title, abstract, full-text review, and same study sample, 15 studies were included for review. Fig. 1 shows the flow diagram of study development. A total of 9 studies involving 765 participants were used in the meta-analysis.

### Characteristics of included studies

Table 1 shows the main characteristics of the included studies. Four studies involved only sinus surgeries, eight involved only septal surgeries, and three involved sinus surgeries, septal surgeries, as well as a combination of the two. One study^8^ used fentanyl as topical anesthetic agent, five studies compared multiple anesthetics to placebo, and ten studies evaluated the effects of a single anesthetic agent. Ten studies provided information regarding postoperative pain reduction, of which seven^8^, ^9^, ^10^, ^11^, ^12^, ^13^, ^14^ were RCTs providing complete data and were included for statistical analysis. Seven studies evaluated pain upon packing removal, only two^15^, ^16^ of which were RCTs with complete data included for statistical analysis. Three^8^, ^16^, ^17^ studies provided information regarding anxiety. All of the studies included for statistical analysis were randomized controlled trials with placebo (Saline, Vaseline, or liquid paraffin) nasal packing as the control group.

### Quality assessment

The risk of bias summary and graph are presented in the supplementary files. More than half of the studies included for review generated unclear to high risk of bias in random sequence generation and allocation concealment. With regard to the blinding of participants and personnel, five studies showed unclear and one showed high risk of bias. Eight studies reported successful methods of outcome assessor blinding. Most studies reported adequate description for incomplete results except for five studies, and all studies were unclear in risk for reporting bias due to lack of study protocol. The quality of our included studies varied greatly and presented moderate to high risks of bias.

### Outcome measures

#### Overall post-operative pain

**Figure 2:**
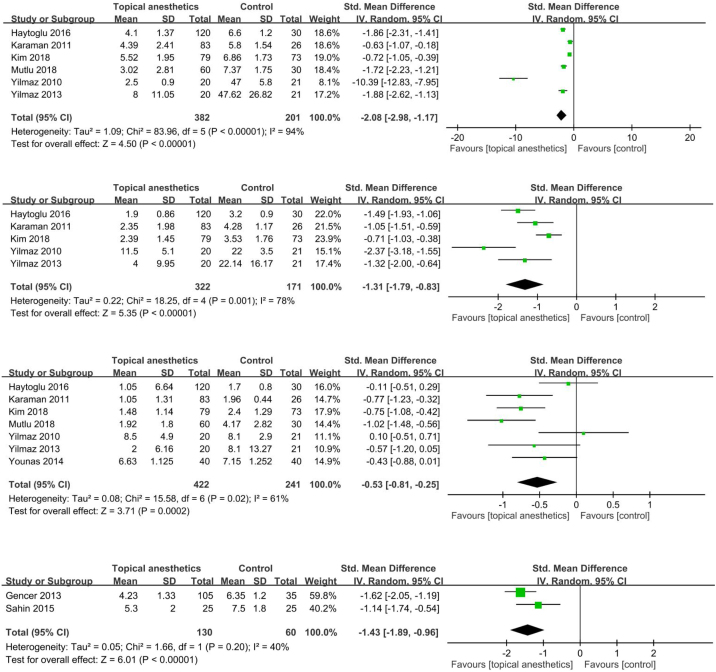
Pain after surgery. (a) 1 h after surgery; (b) 12 h after surgery; (c) 24 h after surgery; (d) upon nasal packing removal.

There were significant SMDs between topical anesthetic-infiltrated nasal packing and placebo packing in favor of the anesthetic group at 1 h (SMD = −2.08; 95% CI −2.98 to −1.17; I^2^ = 94%), 12 h (SMD = −1.31; 95% CI −1.79 to −0.83; I^2^ = 78%), and 24 h (SMD = −0.53; 95% CI −0.81 to −0.25; I^2^ = 61%) after operation (Fig. 2a–c).

#### Pain during nasal packing removal

There was a significant SMD between topical anesthetic-infiltrated nasal packing and placebo packing in favor of the anesthetic group (SMD = −1.43; 95% CI −1.89 to -0.96; I^2^ = 40%) (Fig. 2d).

#### Post-operative pain in sinus surgeries

**Figure 3:**
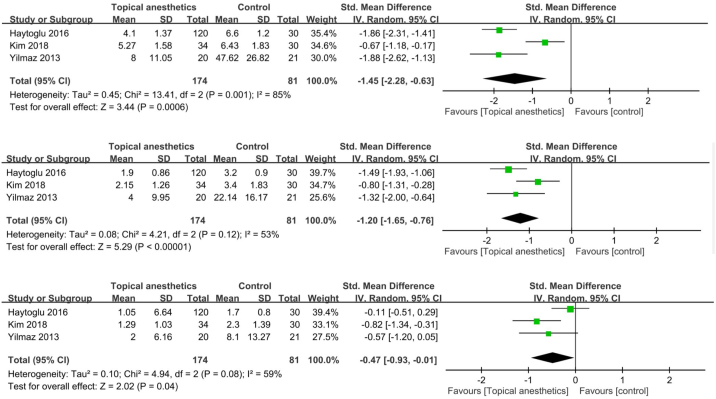
Pain after sinus surgeries. (a) 1 h after surgery; (b) 12 h after surgery; (c) 24 h after surgery.

There were significant SMDs between topical anesthetic-infiltrated nasal packing and placebo packing in favor of the anesthetic group at 1 h (SMD = −1.45; 95% CI −2.28 to −0.63; I^2^ = 85%), 12 h (SMD = −1.2; 95% CI −1.65 to −0.76; I^2^ = 53%), and 24 h (SMD = −0.47; 95% CI −0.93 to −0.01; I^2^ = 59%) after operation (Fig. 3a–c).

#### Anxiety during packing removal

Only 1 RCT^16^ provided complete data for analysis, thus only a qualitative assessment was performed. Sahin et al. conducted a RCT comparing lidocaine with placebo infiltration of nasal packing and found that both the lidocaine group (*p* = 0.647) and placebo (*p* = 0.766) group showed non-significant within-group decreases on the Hamilton anxiety scale after infiltration. No data regarding the difference between the lidocaine and placebo group regarding anxiety were provided.^16^ Apuhan et al. compared levobupivacaine and prilocaine to saline packing in the contralateral nostril, and found both anesthetics groups showed statistically significant improvements on the Ramsay Sedation Scale during infiltration, nasal packing removal, and 30 min after nasal packing removal compared to the saline group. No significant differences were observed between the two anesthetic groups at the above-mentioned time.^17^ Kim et al. used fentanyl as the infiltrating agent and found that the fentanyl group scored a significantly higher mean on the Ramsey sedation scale compared to control in patients receiving ESS and ESS + septoplasty, but did not specify the RSS at different postoperative hours.^8^

#### Postoperative bleeding

Three studies evaluated postoperative bleeding. Mo et al. found that patients receiving bilateral Functional Endoscopic Sinus Surgery (FESS) with lidocaine infiltration of NasoPore (absorbable) sinus packing had significantly less bleeding at the 8th (*p* = 0.026) and 24th (*p* = 0.002) postoperative hours.^18^ Karaman et al. compared lidocaine + adrenaline, bupivacaine and ropivacaine infiltration of Merocel nasal packing (non-absorbable) to saline infiltration and found no significant difference (*p* > 0.05) between study groups and control group.^13^ Mutlu et al. compare lidocaine + adrenaline, tetracaine and articaine + adrenaline infiltration of Merocel nasal packing to saline infiltration in patients receiving septoplasty, and found that only the articaine group showed significantly decreased (*p* < 0.05) postoperative hemorrhage.^11^

### Adverse events

Three studies documented adverse events, none of which reported significant adverse events.^8^, ^11^, ^18^ Two studies measured postoperative blood pressure changes and heart rate fluctuations,^8^, ^18^ both of which reported no differences in postoperative blood pressure changes. Less heart rate fluctuations were observed in the anesthetics group in both studies.

## Discussion

This review investigated the effectiveness of local anesthetics-infiltrated nasal packing compared to placebo nasal packing regarding pain and anxiety reduction after sinonasal surgeries. The methodological quality of our included studies varied greatly from moderate to high risks of bias. Our study suggested that the use of local anesthetics-infiltrated nasal packing significantly reduced pain 1 h, 12 h and 24 h after surgery, for all surgeries combined or in sinus surgeries, as well as upon nasal packing removal. As for anxiety, postoperative bleeding, and adverse events, only a small number of trials were available for qualitative review but were generally in favor of local anesthetics-infiltrated nasal packing. Considering the significant risks of bias present in methodology of most studies, the present study provided low level of evidence to support the use of local anesthetics-infiltrated nasal packing in managing pain after nasal surgeries.

To our knowledge, this is the first meta-analysis evaluating the effect of adding local-anesthetics to nasal packing in all surgeries of the nasal cavity. A Cochrane review in 2018 evaluated the effects of perioperative local anesthesia on reducing pain following septal surgery.^7^ They found that adding local anesthetics to nasal packing significantly reduced pain at 12 h (MD = −16.95, 95% CI −22.27 to −11.62, involving 2 RCTs) and 24 h (MD = −7.35, 95% CI −9.76 to −5.29, involving 4 RCTs) postoperatively, and decreased the need for additional analgesia (OR = 0.15, 95% CI 0.07 to 0.34, involving 2 RCTs). The quality of evidence for outcome regarding pain was deemed low to very low by the authors, and the study did not include studies involving surgeries of sinuses or the lateral nasal wall. The present study provided further evidence that adding local anesthetics to nasal packing significantly reduced pain in all surgeries of the nasal cavity. Generally speaking, all of our included studies significantly favored anesthetics infiltration of nasal packing regarding pain reduction. However, the effect was not uniform across all studies at 24 h. It should be noted that similar to the Cochrane review, a great number of our included studies showed significant risk of bias in terms of methodological quality, so the results should be interpreted with caution.

In terms of anxiety reduction, our study provided mixed results. Only three studies were available for qualitative analysis, one of which did not favor anesthetic infiltration. Of the remaining two in favor of anesthetic infiltration, one did not report complete data. It should also be noted that although RSS contains the description of anxiety, it is an evaluation of the level of sedation, thus is not a direct measurement of anxiety. Our review provides very low evidence for the effect of topical anesthetics on reducing anxiety, and further studies are needed.

Regarding bleeding and adverse events, a review of our included studies did not demonstrate significant side effects, and several studies report less postoperative bleeding compared to placebo. However, as these were not the primary outcomes of interest, only a few of our included studies provided data regarding this issue. Several previous studies have demonstrated the potential of nasal packing for adverse events. A study conducted by Zayyan et al. reported that nasal packing, whether totally occlusive or with a patent airway, significantly increased minimum heart rate, decreased maximum heart rate, and resulted in increased heart rate variability. They proposed that these changes were related to the vagal response.^19^ Banglawala et al. conducted a systemic review to investigate the cardiopulmonary impact of bilateral nasal packing and reported no significant adverse cardiopulmonary changes.^20^ However, their conclusion was largely based on arterial blood gas parameters and based on studies of varying quality, so their results should be interpreted with caution. Therefore, we believe nasal packing with or without local anesthetics should still be used cautiously based on patients’ individual comorbidities and risks.

Although nasal packing is not performed routinely in all sinonasal surgeries, the present study provides evidence that adding local anesthetics to nasal packing is an effective method to reduce pain in patients requiring nasal packing. This potentially has implications in managing patients with significant pain following nasal packing. However, given the low level of evidence of the present study and the uncertainty regarding side effects, clinicians should still carefully weigh the potential benefits of nasal packing against risks. Since our results are based on studies of varying methodological quality, and more high-quality trials are needed.

### Limitations

Several limitations should be addressed. First, included studies differed in study design, study population, infiltrating agents, and outcome measures, potentially contributing to heterogeneity. Second, only a limited number of RCTs were available for analysis regarding several outcomes, resulting in a lack of power. Third, a great number of included studies had significant risk of bias in methodology. Finally, we did not differentiate between various topical anesthetic agents.

## Conclusion

Our study supports anesthetics infiltration of nasal packing as an effective method in managing pain in patients with nasal packing after sinonasal surgeries. However, the level of evidence is low. More high-quality randomized controlled trials are needed to establish its effectiveness in reducing anxiety.

## Conflicts of interest

The authors declare no conflicts of interest.

## References

[1] Kim J.S., Kwon S.H. (2017). Is nonabsorbable nasal packing after septoplasty essential? A meta-analysis. Laryngoscope.

[2] Stern-Shavit S., Nachalon Y., Leshno M., Soudry E. (2017). Middle meatal packing in endoscopic sinus surgery-to pack or not to pack?-a decision-analysis model. Laryngoscope.

[3] Vlastarakos P.V., Iacovou E., Fetta M., Tapis M., Nikolopoulos T.P. (2016). How effective is postoperative packing in FESS patients? A critical analysis of published interventional studies. Eur Arch Otorhinolaryngol.

[4] Halderman A.A., Sindwani R., Woodard T.D. (2015). Hemorrhagic complications of endoscopic sinus surgery. Otolaryngol Clin North Am.

[5] Adriaensen G., Lim K.H., Fokkens W.J. (2017). Safety and efficacy of a bioabsorbable fluticasone propionate-eluting sinus dressing in postoperative management of endoscopic sinus surgery: a randomized clinical trial. Int Forum Allergy Rhinol.

[6] Akiyama K., Karaki M., Yonezaki M., Goto R., Inamoto R., Hoshikawa H. (2014). Usefulness of nasal packing with silver-containing carboxy methylated cellulose in endonasal sinus surgery. Auris Nasus Larynx.

[7] Fujiwara T., Kuriyama A., Kato Y., Fukuoka T., Ota E. (2018). Perioperative local anaesthesia for reducing pain following septal surgery. Cochrane Database Syst Rev.

[8] Kim K.S., Yeo N.K., Kim S.S., Park W.S., Kwak S.H., Cho S.H. (2018). Effect of Fentanyl Nasal Packing treatment on patients with acute postoperative pain after nasal operation: a randomized double-blind controlled trial. Ann Otol Rhinol Laryngol.

[9] Yilmaz S., Kocaman Akbay B., Yildizbas S., Guclu E., Yaman H., Yalcin Sezen G. (2010). Efficacy of topical levobupivacaine in control of postoperative pain after septoplasty. J Otolaryngol Head Neck Surg.

[10] Younas M. (2014). Efficacy of 5% lignocaine ointment in reducing the post-operative pain due to intra-nasal packs. Med Forum Mon.

[11] Mutlu V., Kaya Z. (2018). Comparison of the effect of the lidocaine, tetracaine, and articaine application into nasal packs on pain and hemorrhage after septoplasty. Eur Arch Otorhinolaryngol.

[12] Yilmaz S., Yildizbas S., Guclu E., Yaman H., Yalcin Sezen G. (2013). Topical levobupivacaine efficacy in pain control after functional endoscopic sinus surgery. Otolaryngol Head Neck Surg.

[13] Karaman E., Gungor G., Alimoglu Y., Kilic E., Tarakci E., Bozkurt P. (2011). The effect of lidocaine, bupivacaine and ropivacaine in nasal packs on pain and hemorrhage after septoplasty. Eur Arch Otorhinolaryngol.

[14] Haytoğlu S., Kuran G., Muluk N.B., Arıkan O.K. (2016). Different anesthetic agents-soaked sinus packings on pain management after functional endoscopic sinus surgery: which is the most effective?. Eur Arch Otorhinolaryngol.

[15] Gencer Z.K., Ozkiris M., Gencer M., Saydam L. (2013). Comparison of ropivacaine, bupivacaine, prilocaine, and lidocaine in the management of pain and hemorrhage during nasal pack removal. Am J Rhinol Allergy.

[16] Sahin C., Aras H.I. (2015). Effect on patient anxiety of lidocaine infiltration into nasal packing after septoplasty: prospective, controlled study. J Laryngol Otol.

[17] Apuhan T., Yildirim Y.S., Gulcu N., Kocoglu H., Karagoz Y. (2011). The effect of prilocaine or levobupivacaine infiltration on pain during nasal packing removal. Otolaryngol Head Neck Surg.

[18] Mo J.H., Park Y.M., Chung Y.J. (2013). Effect of lidocaine-soaked nasal packing on pain relief after endoscopic sinus surgery. Am J Rhinol Allergy.

[19] Zeyyan E., Bajin M.D., Aytemir K., Yilmaz T. (2010). The effects on cardiac functions and arterial blood gases of totally occluding nasal packs and nasal packs with airway. Laryngoscope.

[20] Banglawala S.M., Gill M.S., Dhillion N., Khan J.S., Gupta M.K., Psaltis A. (2014). Nasal packing after septoplasty: cardiopulmonary impact. JAMA Otolaryngol Head Neck Surg.

